# Laboratory automation: changing the role of the technical professional

**DOI:** 10.1155/S1463924693000045

**Published:** 1993

**Authors:** Kevin J. Halloran

**Affiliations:** Wallace Laboratories Carter-Wallace Inc. Half Acre Road Cranbury New Jersey 08512 USA


Journal of Automatic Chemistry, Vol. 15, No. 1 (January–February 1993), pp. 13–17

### Laboratory automation: changing the role of the technical professional

**Kevin J. Halloran**
*Wallace Laboratories, Carter-Wallace Inc., Half Acre Road, Cranbury, New Jersey 08512, USA*

### Robots in industry

During the last two decades, robots have gone from exotic to commonplace in the manufacturing industries. As companies strive to gain competitive advantage, mechanization of repetitive tasks has been a primary means of increasing productivity and reducing cost. The impact of robotization is now affecting the world economy [1]. While not the first to develop robotics, Japan has successfully applied the technology to compensate for labour shortages, to increase productivity, and to develop quality products through standardization [2]. The Japanese productivity gains have, to a large extent, been realized through the substitution of capital equipment for human labour [3].

As robotics spread through the manufacturing sector, there has been heated debate concerning the impact of automation on the worker [4–6]. The technologist views robotics as a means of creating high-quality jobs and improving the quality of life [7]. Workers, however, often perceive automation as a threat to their job security [8] – researchers report that workers feel more threatened by robotics than any other form of automation [9] and it is believed that workers feel that a robot often directly displaces a worker.

### Robots in the laboratory

Similarly, robots began to appear in the analytical chemistry laboratory during the early 1980s. The same reasoning behind the need for automation in manufacturing can also be applied to the laboratory – productivity can be dramatically increased by the automation of routine testing. The analytical chemistry laboratory tests a large number of samples using standardized procedures, while maintaining a quick turnaround time. Many of the tasks that are performed in the laboratory are routine and highly repetitive. Interestingly, studies have shown that the quality of work performed by laboratory workers decreases as the volume of work increases [10].

Employee perceptions concerning laboratory automation range from enthusiasm to apprehension. The laboratory manager and the non-Ph.D. scientists are usually receptive to the technology. Technicians, on the other hand, may be reluctant supporters and may even feel threatened by the technology. Employee fears that automation will displace laboratory workers have so far been unsubstantiated. Robot manufacturers promote the image that automation will free the scientist to perform more innovative tasks [11]. Several pharmaceutical companies have announced that laboratory automation will not displace workers in their organizations [12, 13]; in fact, there are reports that automation actually causes increases in the laboratory staff [14].

The author's staff has increased over the years, in spite of the introduction and growth of robotics. As the throughput capabilities of the laboratory was increased by automation, the workload dramatically increased, thus robotics allowed the laboratory to keep abreast of a spiralling workload.

It is easy to see how management could develop the perception that a robot is an unlimited resource and an immediate cure for an understaffed laboratory. unfortunately, robots are not a cure for a general lack of manpower. In fact, robots require people to set them up and maintain them. With this misconception, the laboratory manager may be pressured to accept shorter and shorter deadlines. A question that is often put forward at staff meetings is: 'Why can't you just put it on the robot?' Ultimately, management could become disappointed when the robot does not live up to their unrealistic expectations. The laboratory manager must educate his peers and superiors about the limitations of robotics and the resources required to develop and validate an automated method, while at the same time, demonstrating the advantages of the technology.

Do robots free laboratory workers to perform more innovative projects? That depends. In most laboratories, the methods that are automated are analyses normally performed by either a technician or a non-Ph.D. scientist. The assignments performed by Ph.D. scientists are rarely candidates for automation because of their complexity and uniqueness. The employee who develops the robotic system, often a non-Ph.D., is freed from repetitive work by the very nature of the project. Other non-Ph.D. scientists may be given non-routine work, provided that they have the skills necessary for more advanced assignments. However, unless the manager changes the way work is assigned, the technician will not benefit from automation. Low volume routine analyses will continue to be performed manually. The assay that was automated is simply replaced by another routine job. Laboratory managers can do better than this: all laboratory workers should benefit from automation, but this needs careful planning, technical training, and skill development.

This paper was read at the International Symposium on Laboratory Automation and Robotics (October 1992, Boston, USA).

© Zymark Corporation 1993

### New roles in the laboratory

The growth of automation in the analytical laboratory will require changes in some job functions. William Godolphin has proposed that the implementation of robotics provides an opportunity to re-skill the laboratory, suggesting that management should provide skill training in mechanics, electronics and computers [15]. Erich Bloch has suggested that in order for a company to take full advantage of a new technology, continually upgrading the skills of its employees is just as important as meeting deadlines [16]. Educational programmes should be open to all laboratory personnel including technicians; as technicians develop new skills, they should feel less threatened by automation.

The spread of automation has crated new positions in the laboratory. Frank Zenie has proposed the appointment of an 'automation specialist' who will manage the development of new robotic applications [17]. Since this position does not require a Ph.D., it will provide an opportunity for either a non-Ph.D. scientist, or possibly a technician. Some organizations have taken this idea further and organized automation development groups [18].

### Worker roles and robots

In the analytical chemistry laboratory there are four main types of workers: the technician, the non-Ph.D. scientist, the bench Ph.D. scientist, and the laboratory manager. As laboratory automation spreads, the roles played by each of these workers will evolve.

The following discussions propose strategies for integrating automation into the laboratory, as well as applying the technology to enrich the careers of laboratory workers. The characteristics described herein for each employee group are common traits that the author has observed over a 14-year career spanning four pharmaceutical organizations. Of course, not every employee will match the respective group profile. In addition, companies vary in their laboratory work environments and career development opportunities.

### The technician

A typical laboratory technician is an employee who does not have a four-year college degree in a scientific discipline. Technicians are expected to perform routine analytical testing using well established procedures; their work is highly repetitive. Frequently, technicians develop an expertise with their set of analyses that cannot be matched by the scientists in the laboratory. Some technicians may not see themselves as part of the laboratory team. They usually do not participate in project planning, 'brainstorming' or strategy discussions. As a result, they may not be motivated to provide suggestions for improvements in the laboratory or the handling of new projects. Technical management researchers sensibly propose that making technicians true members of the technical team is critical to the success of a project [19]. This can be a very difficult task, but integrating technicians as members of the laboratory team is an important aspect of technical management that should not be neglected.

Some laboratory technicians are apprehensive toward laboratory robotics. This apprehension can create a barrier that may make it difficult to rally technicians behind laboratory automation. In this era of consolidation within the pharmaceutical industry, one can see why some technicians could perceive a newly installed laboratory robot as a threat to their employment.

Involving technicians early on in robotics will help to dissipate any anxieties that they may have; in the author's laboratory, they are trained to operate the robots. A technician is often the end-user of a robotic system. A word of caution; the laboratory manager must be careful not to overload the robot end-user. If the analyst is overwhelmed with samples, a resentment of automation could occur.

If managed properly, technicians will strongly benefit from the introduction of robotics in their laboratory. When the technician is assigned 'ownership' to maintain a robot and plan its work schedule, new pride, self-worth and a feeling of value to the organization may develop. The technician will become the administrator of a significant resource. As technicians become more familiar with the equipment, they should be given an opportunity to develop new applications. As robot end-users, the technicians become more involved in the day-to-day operation of the laboratory.

### *The non-Ph.D. scientist*

The non-Ph.D. scientist has either a bachelor's or master's degree in a scientific discipline. On many days, they perform the same analyses as the technicians. Unlike technicians, non-Ph.D.s are also assigned investigative projects where they work independently or with other scientists. The more experienced or higher educated the non-Ph.D. scientist, the more advanced the work assignment.

A large number of non-Ph.D. scientists who start their careers in the laboratory, will eventually leave. The routine workload may be a factor – these employees may also perceive that they face a finite career path and limited opportunities if they stay in the laboratory. The pharmaceutical laboratory environment is very degree oriented – without an advanced degree it can be difficult to advance beyond a certain level. The ambitious non-Ph.D. scientist is compelled to earn an advanced degree or make a career move into another area of the company. Finally, there is the issue of visibility – by its very nature, routine work is not visible. Non-Ph.D. employees may perceive that people in other areas of the company with their educational background are more visible to management and appear to be on long-term career paths. On the other hand, the non-Ph.D. employee who stays in the laboratory will very likely hit a career plateau [20].

The non-Ph.D. scientist is the laboratory manager's best resource when implementing laboratory automation.

Studies have shown that the employee selected most often to design a laboratory robotic system is a self-motivated non-Ph.D. scientist [21]. Many non-Ph.D. scientists actively campaign for robot development projects. This is because robots can be very visible – robots provide an opportunity to gain visibility within and outside the organization through publications and presentations. Also, robotics provides an opportunity for the employee to assume 'ownership' over a non-routine, innovative, developmental project – the employee will enjoy personal satisfaction that comes with the successful completion of the project. Finally the employee receives positive psychological rewards through special recognition from the laboratory manager and higher level managers, as well as other people within the organization.

The growth of robotics supports the appointment of an 'automation specialist'. The automation specialist's responsibilities include: interacting with equipment manufacturers, developing new applications, training of personnel, and implementing control systems. As an increasing number of methods are automated, a critical mass of automated methods justifies the formation of an automation group. This automation group will require the appointment of a group leader. Both the automation specialist and the automation group leader positions provide new opportunities within the laboratory for non-Ph.D. scientists. Since there is a natural progression from robotics to computers, some employees may eventually evolve into laboratory computer specialists, leading to new technical careers.

As automation reduces the number of routine assignments performed manually, there is an opportunity to upgrade the work assignments of those non-Ph.D. scientists who are not involved with robotics. These employees can now be directed toward method development or investigational assignments, either working independently or under the wing of a senior scientist. With careful management, a mentoring system could develop. This mentoring system is one element of a technical training programme that is needed to develop the junior level chemists and provide them with the tools they will need to perform more advanced assignments. Given more challenging assignments, visibility and technical growth, non-Ph.D. employees may be less motivated to transfer out of the laboratory.

### *The bench Ph.D. scientist*

For the most part, bench Ph.D. scientists who are not responsible for high volumes of repetitive analyses are not heavily involved with laboratory robotics. However, analytical methods are often developed by Ph.D. scientists and later transferred into the analytical support laboratory. Method developers must keep automation in mind when they develop new methods. They must be familiar with the constraints of robotics in order to develop methods that are not only suitable for manual sample preparation, but are also easily transferred to automation equipment. Quick, efficient automation of manual methods is particularly critical in the research laboratory, where there is a finite life to the steady, high volume stream of analyses for a given product. Substantial laboratory productivity is lost because high volume sample analyses can not be transferred to robotic hardware without significant modifications to the analytical methodology or customization of the hardware.

Since bench Ph.D.s are usually not involved with robotics, the tangible effect of automation on these scientists is not evident. The intangible effects may be quite significant. As non-Ph.D. scientists are freed from routine work, they are available to work with the Ph.D.s on research teams. More human talent and effort will be directed toward the more complex and innovative projects. With more talent focused on research projects, management will expect greater productivity and faster completion rates from their research laboratories.

The role of the bench Ph.D. scientist may change. Traditionally, bench Ph.D. scientists work independently. Now they will lead small technical teams. They will spend more of their time planning work strategies and mentoring their coworkers. To be effective, Ph.D. scientists must possess good strategic planning and interpersonal skills. These skill groups may be underdeveloped, since management skills are often neglected in technical education [22]. The laboratory manager should propose and encourage career enrichment plans that will develop the project planning and interpersonal skills of senior scientists.

### *The laboratory manager*

The laboratory manager is the primary beneficiary of laboratory automation. The laboratory manager's performance evaluation is based, in part, on the level of productivity sustained by the laboratory. Productivity issues may even be reduced to hard statistics: number of samples tested during a given period, the mean turnaround time for a sample analysis etc. On the other hand, productivity measures could be more subjective; is the manager routinely late meeting deadlines?

Potentially, the laboratory manager has much to gain from automation. The gains will be in the form of higher productivity and a greater ability to schedule work [23]. In addition, managers commonly complain that they desire more time to be creative [24]. The use of automation may give them that time. Less evident, automation provides an opportunity to re-energize the workforce, thus boosting employee morale [25].

The laboratory manager who wishes to purchase a robot must be prepared to champion the technology within the organization. By definition, a product champion; 'creates, defines or adopts . . . a new technological innovation and who is willing to risk his or her own position and prestige to make possible the innovation's successful implementation' [26]. Automation equipment requires substantial capital investment. Because of this investment, and their unique visibility, laboratory managers assume more risk by championing robotics than most other forms of laboratory technology. A robot that sits idle is noticed and frowned upon by management. In contrast, a mass spectrometer is just as impressive idle as it is in use. The laboratory manager must assume six roles in order for an active automations program to succeed. These roles are *selecting* the right person to develop the system, *facilitating* the acquisition of resources, *setting* overall and intermediate project goals, *guiding* the project to assure that it remains on track, *insulating* the robot developer from distractions, and *promoting* the success of both the project and the developer. Successful execution by the laboratory manager in all of these areas is critical to the success of a robotics project.

With the purchase of the first robotics system, the laboratory manager sets out to select the person who will develop the first automated methods. The first application is most critical because it may determine the reputation of the technology within the organization for years to come. The manager must carefully choose a person with the right qualities. First, the robot developer should understand the analytical technique to be automated. Preferably, the person should have thorough hands-on experience with the manual method. The person must be self-motivated, creative, and be able to work independently. In addition, the developer should possess good interpersonal skills. Effective 'people' skills are needed in order for the developer to seek out technical resource people both within and outside the company. Finally, the developer should have a genuine interest in robotics and a commitment to its successful implementation. Technical skills, such as a knowledge of computers and electronics, are a bonus.

The laboratory manager must facilitate the acquisition of resources. After the installation of the robot, additional equipment is often needed. Equipment manufacturers continually introduce new products that are distinct improvements over existing hardware. Laboratory managers should develop the ability to rapidly negotiate their company's purchase approval channel in order to quickly procure additional equipment. The manager should also negotiate with equipment manufacturers for 'loaner' or 'evaluation' equipment. To the discredit of the laboratory manager, a robotics project could fail because a capital purchase was tangled in red tape. The robot developer's hands may be tied because he or she was not supplied with the proper equipment.

The laboratory manager is responsible for the timely development of the robotics project. To accomplish this, the manager should make sure that intermediate and overall project goals are established. This does not mean that the manager should take a direct, authoritative approach. The robot developer must be given a level of freedom and creative latitude. The manager and the developer should devise a strategic plan that identifies the project's key stages, or milestones. A completion date should be assigned to each milestone, as well as to the overall project. The manager and the robot developer should revisit the development plan on a regular basis. In general, the manager's task is to assure that the project remains on track. As with any innovator, the developer could easily veer off course or become bogged down with one aspect of the project. By carefully monitoring the project, the manager will detect any change in direction and catch the developer before he or she wanders too far off track.

The laboratory manager must insulate the robot developer from distractions. These distractions could take the form of negative feedback from employees who are apprehensive about automation. Employees who were overlooked when a developer was chosen may also harbor some negative feelings. Then there are those people who mean well, but are simply distracting. These are the individuals who freely, and repeatedly share their opinions on how to best automate the method. Finally, the distraction would come from the laboratory manager who assigns the developer 'little' unrelated projects to be performed during spare time. In any case, it is the responsibility of the manager to provide the developer with quality, undisturbed time to devote all efforts to the automation project.

The last active role played by the laboratory manager is to promote the success of both the project and the robot developer. A failed robot will promote itself. A successful automation project is not automatically visible because routine analytical testing is not visible. The laboratory manager must continually make management aware of the productivity gains realized by using automation equipment and assure them that their money was well spent. In addition, the manager must illuminate the accomplishments of the robot developer. This person desired visibility. Visibility can be gained by allowing developers to demonstrate their systems, encouraging them to present internal seminars, and persuading them to present their work at professional meetings.

A flourishing automation programme will present new challenges for the laboratory manager, for example assuring the continued validity of the data generated by the robot, managing an increase in information caused by the increased productivity, suppressing the tendency to avoid risk by re-analysis, and formulating career enrichment strategies for all employees. The manager must implement a set of controls which assure that the robotic system is functioning as intended and continues to produce valid data. When a worker performs an analysis, it is assumed that the worker is following the test procedure. With laboratory automation, the manager must assume the responsibility for the validity of the data. A series of operational checks should be implemented that will detect equipment wear which could ultimately invalidate the system.

The second challenge to the laboratory manager is caused by an increase in information generated. Automation increases the number of samples tested during a period, and the manager may become overwhelmed if he or she does not have the computer systems in place to handle the increased volume of data. The sample throughput of a robotic system can be hobbled by an inappropriate or inflexible data management system.

Third, the manager must not view automation as a means of avoiding risk. When a test is performed by an analyst, and a variant result is produced, the manager evaluates the situation and makes a judgement concerning the need to repeat the test. If the test is automated, the manager is inclined to repeat it. Productivity gains through automation can be eroded by an increase in needless sample re-analysis.

Finally, the laboratory manager must become more active in skill development. The manager must strive to develop employees in order to re-tool the staff for their new roles in the laboratory. Employees at all levels should be encouraged to attend technical seminars. Technicians should be trained in robotics, first as end-users and then as developers. Those non-Ph.D. scientists who express an interest in automation should be trained in robotics, computers and electronics. Non-Ph.D. workers, in general, must have technical training in order for them to effectively participate in research projects. The interpersonal and project planning skills of the senior scientists must be developed in order to build effective research teams. Finally, the laboratory manager must seek out new non-routine projects to replace the routine testing that has been automated.

### Conclusions

As a management tool, laboratory automation can be a means of enriching all members of the laboratory. It is a resource that, if managed properly, will increase productivity and improve work scheduling, while providing new opportunities to re-skill and develop all laboratory personnel. However, in order to seize these opportunities, the laboratory manager must champion the technology and provide an active programme of technical training and skill development.

### Acknowledgement

I would like to thank Dorothy Martynuk and Daniel Zuccarello for their assistance.

### References

1. Kinoshita, S. and Yamada, M., *Technological Forecasting and Social Change*, **35** (1989).
2. Ishitani, H. and Kaya, T., *Technological Forecasting and Social Change*, **35** (1989).
3. Ullrich, R. A., *The Robotics Primer* (Prentice Hall, Englewood Cliffs, NJ, 1983), p. 5.
4. Guterl, F., *IEEE Spectrum*, **20** (1983) No. 5.
5. Nitzan, D., *IEEE Journal of Robotics and Automation*, **RA-1** (1985), 1.
6. Tchijov, I., *Technological Forecasting and Social Change*, **35** (1989).
7. Jenkin, P., *New Scientist*, **24** (1983), 2.
8. Chao, G. T. and Kozlowski, S. W. J., *Journal of Applied Psychology*, **71** (1986), 1
9. Argote, L., Goodman, P. S. and Schkade, D., *Sloan Management Review*, **24** (1983), 3.
10. Whitlow, K. J. and Campbell, D. J., *American Journal of Clinical Pathologists*, **79** (1983).
11. Curley, J., In *Advances in Laboratory Automation – Robotics*; Strimaitis, J. R. and Hawk, G. L. (Eds), (Zymark Corporation: Hopkinton, MA, 1984).
12. Miller R. G., In *Advances in Laboratory Automation – Robotics*; Strimaitis, J. R. and Hawk, G. L. (Eds), (Zymark Corporation, Hopkinton, MA, 1985).
13. Hamilton, S. D., In *Advances in Laboratory Automation – Robotics*; Strimaitis, J. R. and Little, J. N. (Eds), (Zymark Corporation, Hopkinton, MA, 1990).
14. Taylor, G. L., Smith, T. R. and Kamla, G. J., In *Advances in Laboratory Automation – Robotics*; Strimaitis, J. R. and Little, J. N. (Eds). (Zymark Corporation, Hopkinton, MA, 1990).
15. Godolphin, W., In *Advances in Laboratory Automation – Robotics*; Strimaitis, J. R. and Hawk, G. L. (Eds), (Zymark Corporation, Hopkinton, MA, 1989).
16. Bloch, E., *Research Management*, **27** (1984), 6.
17. Zenie, F. H., In *Advances in Laboratory Automation – Robotics*; Strimaitis, J. R. and Hawk, G. L. (Eds), (Zymark Corporation, Hopkinton, MA, 1984).
18. Hamilton, S. D., op. cit., p. 91.
19. Jain, R. K. and Triandis, H. C., *Management of the R&D Organization* (John Wiley & Sons, New York, 1990).
20. Ference, T. P., Stoner, J. A. F. and Warren, E. K., *Academy of Management Review*, **2** (1977).
21. Zenie, F. H., In *Advances in Laboratory Automation – Robotics*; Strimaitis, J. R. and Hawk, G. L. (Eds), (Zymark Corporation: Hopkinton, MA, 1989).
22. Badawy, M. K., In *Managing Professionals In Innovative Organizations*; Katz, R. (Ed), (Ballinger Publishing Company, New York, NY, 1988).
23. Hurst, W. J. and Mortimer, J. W. *Laboratory Robotics* (VCH Publishers, New York, 1987).
24. Tagiuri, R., *Research Management*, **28** (1985), 5.
25. Curley, J., In *Advances in Laboratory Automation – Robotics*; Strimaitis, J. R. and Hawk, G. L. (Eds), (Zymark Corporation, Hopkinton, MA, 1985).
26. Maidique, M. A., *Sloan Management Review*, **21** (1980), 64.

